# Acute lower leg transcutaneous electrical nerve stimulation increases leg blood flow and exercise capacity in patients with peripheral arterial disease

**DOI:** 10.1113/EP093618

**Published:** 2026-07-21

**Authors:** Thomas K. Pellinger, Catherine B. Neighbors, Joshua C. Hefta, Patrick H. Luo, Carl B. Suarez, Erotokritos T. Varlas, Grant H. Simmons, Brett J. Wong

**Affiliations:** ^1^ Department of Physical Therapy University of Maryland Eastern Shore Princess Anne Maryland USA; ^2^ Sentara Center for Healthcare Simulation and Immersive Learning Virginia Health Sciences at Old Dominion University Norfolk Virginia USA; ^3^ Department of Medical Pharmacology & Physiology University of Missouri Columbia Missouri USA; ^4^ Department of Kinesiology & Health Georgia State University Atlanta Georgia USA

**Keywords:** claudication, electric stimulation therapy, six‐minute walk test

## Abstract

We tested the hypothesis that two different durations of lower leg transcutaneous electrical nerve stimulation (TENS) increase post‐TENS popliteal artery blood flow (PABF), popliteal vascular conductance (PVC), and 6‐min walk distance (6MWD) in patients with peripheral artery disease (PAD). Seven subjects (5 male, 2 female) with PAD (67.9 ± 7.4 years; claudication: ankle‐brachial index (ABI) <0.90) participated in three randomized treatment sessions: control or bilateral TENS (3.0 Hz burst mode, 250 µs) for either 15 or 45 min. PABF (Doppler ultrasound) was measured before and after treatment. 6MWD and time/distance to claudication onset were measured 25 min post‐treatment during each session. PABF, PVC and 6MWD increased after both 15 and 45 min of TENS (*P* < 0.05, post‐TENS vs. pre‐TENS baseline/control). Our preliminary results suggest that as little as 15 min of lower leg TENS increases leg blood flow and exercise capacity in individuals with PAD.

## Abstract (highlights)

- **What is the central question of this study?**
  Can a single bout of lower leg transcutaneous electrical nerve stimulation (TENS), for either 15 or 45 min, improve both post‐TENS leg perfusion and walking performance in patients with peripheral artery disease (PAD)?
- **What is the main finding and its importance?**
  As little as 15 min of lower leg TENS improves both post‐TENS leg perfusion and 6‐min walk distance in patients with PAD, highlighting a safe, affordable intervention to improve exercise tolerance in this population.

## 1 INTRODUCTION

Peripheral arterial disease (PAD), which affects over 230 million adults worldwide (Song et al., 2020) and approximately 8–10 million Americans (Creager et al., 2021), is characterized by occlusive plaques in the arteries that perfuse the legs (Criqui et al., 2021). This reduction in lower leg perfusion results in the hallmark sign of PAD, intermittent claudication (IC): lower extremity pain and fatigue with physical exertion that is relieved with rest (Aday & Matsushita, 2021). People with PAD often have greater functional impairment and faster mobility loss than those without PAD (McDermott, 2017), which leads to an inactive lifestyle and increased risk of cardiovascular mortality (Hamburg & Creager, 2017). These patients may undergo revascularization to restore blood flow and improve function, but it is invasive, costly and poses more complications while providing comparable benefits to supervised exercise (Fakhry et al., 2018). Exercise therapy has been shown to improve overall function, as well as walking performance and quality of life in individuals with PAD by increasing perfusion and delaying onset of claudication (Gornik et al., 2024; McDermott, 2017; Meneses et al., 2018), but adherence to exercise is often challenging for this population (Barbosa et al., 2015; Bartelink et al., 2004). Therefore, it is important to identify practical interventions to improve exercise tolerance and encourage a more active lifestyle in patients with PAD.

Transcutaneous electrical nerve stimulation (TENS) offers a potential non‐invasive treatment option for this patient population that may be employed either independently by patients or under the supervision of a healthcare provider. Previous investigations indicate that acute administration of TENS can improve functional capacity in patients with PAD (Labrunée et al., 2015; Seenan et al., 2016). There is evidence to suggest that TENS administration may achieve these improvements by blunting pain (Mendell, 2014; Patel et al., 2025) and/or increasing limb blood flow (Debreceni et al., 1995; Jéhannin et al., 2020; Kaada, 1982; Labrunée et al., 2013). However, the data supporting the efficacy of TENS administration on patients with PAD are still limited. Moreover, access to the required equipment and/or skills may be limited, as TENS is not commonly utilized in cardiovascular rehabilitation clinics. Therefore, further evidence is needed to increase awareness and support the widespread application of TENS in the treatment of individuals with PAD.

Although TENS has been examined as a potential intervention to improve function in patients with PAD, to our knowledge, no investigation has examined the effect of different durations of TENS on both leg blood flow and walking performance in this population. The purpose of this pilot study was to test the hypothesis that a single bout (of 15 and/or 45 min) of TENS applied to the lower legs will increase resting popliteal artery blood flow (PABF), popliteal vascular conductance (PVC) and 6‐min walk distance (6MWD) in patients with PAD.

## 2 METHODS

### 2.1 Ethical approval

The institutional review board of the University of Maryland Eastern Shore approved this study (Protocol #08‐2021‐001). Each participant gave informed written consent before enrolling in the study and this study conformed to the guidelines set forth in the *Declaration of Helsinki*, and the study was registered on ClinicalTrials.gov (Protocol ID 11112015‐2).

### 2.2 Participants

Seven participants (5 males, 2 female) with PAD (Claudication Fontaine Stage II) aged 67.9 ± 7.4 years volunteered for this study. Inclusion criteria included resting ankle–brachial index (ABI) < 0.90, free of severe exercise limitations due to co‐morbidity, and body mass index (BMI) ≤ 35 kg/m^2^. Subject anthropometric characteristics were as follows: height 172.4 ± 14.6 cm, weight 86.7 ± 21.9 kg, and BMI 28.6 ± 4.0 kg/m^2^. Of the seven participants, four were taking an ACE inhibitor, four were taking a statin, two were taking a beta‐adrenergic receptor antagonist, two were taking an anticoagulant, two were taking metformin, one was taking a calcium channel blocker, and one was taking a diuretic. For all study visits, participants reported to the laboratory at least 3 h postprandial, having refrained from alcohol consumption and exercise for 24 h and consumption of caffeine for 12 h. Participants were instructed to take medications normally, as prescribed by their physicians.

### 2.3 Experimental protocol

Participants reported to a temperature‐controlled laboratory (21–22°C) for parallel experiments on three separate, randomized study visits, each separated by 2–7 days. On each study day, after 10 min of quiet rest, baseline measurements of single‐leg PABF (Doppler ultrasound; Esaote MyLab Sigma, Genoa, Italy), heart rate (HR) and arterial pressure (automated oscillometric device; Welch Allyn Vital Signs Monitor, Skaneateles Falls, NY, USA) were taken. Blood flow was measured on each participant's most affected leg, as indicated by the ABI measurements furnished by his/her physician and confirmed by the participant's subjective report of signs and/or symptoms. For patients with no reported differences between legs, blood flow in the right leg was measured.

Mean blood velocities and diameters of the popliteal artery were measured using a linear ultrasound probe (Esaote L4‐15 linear probe). The entire width of the artery was insonated with an angle of 60°, and velocity measurements were taken immediately before diameter measurements. All measurements were made by the same experienced investigator. PABF was calculated as artery cross‐sectional area multiplied by popliteal mean blood velocity and reported as mL min^−1^. PVC was calculated as PABF/mean arterial pressure and expressed as mL min^−1^ mmHg^−1^. ABI was calculated by dividing the higher systolic pressure of the dorsalis pedis and tibialis posterior vessels at the ankle with the higher of the systolic pressures measured in the brachial artery in both arms (Casey et al., 2019). Blood pressure and heart rate were measured in duplicate. Analysis of data was conducted blind to the experimental conditions.

After baseline measurements, participants moved to a seated position to undergo bilateral TENS for either 15 or 45 min or 15 min of quiet rest (control visit). The 45‐min duration of TENS was based on the current guidelines for supervised exercise therapy for patients with PAD (Gornik et al., 2024). The 15‐min duration of TENS was included as a practical alternative to the 45‐min session, to which patients with PAD might be more adherent (Pellinger et al., 2019). Lower leg TENS (LG‐TEC Dual Combo TENS Unit; LG Med Supply, Cherry Hill, NJ, USA) was conducted via placement of electrodes to the motor points of each gastrocnemius muscle using a burst mode frequency of 3.0 Hz and pulse duration of 250 µs. Intensity was increased in increments of 10 mA approximately every 10 s to produce a pain free and visible muscle contraction. Immediately after TENS (or control), participants returned to the supine position and after 10 min of rest, measurements of PABF, heart rate, arterial pressure and ABI were taken. After these measurements (25 min after completion of TENS or period of quiet rest on control visit), participants performed a self‐paced 6‐min walk test (ATS Committee on Proficiency Standards for Clinical Pulmonary Function Laboratories, 2002). The timing of the post‐TENS/control measurements and subsequent 6MWT was chosen to determine if the effects of TENS were sustained long enough to support practical improvements in walking capacity for patients with PAD. Subjective ratings of perceived exertion, claudication and dyspnoea were collected each minute during the walk. In addition, participants were asked to report when they first experienced claudication symptoms, if applicable. For both study visits, participants were blinded to the distance completed during the 6‐min walk test.

### 2.4 Statistics

Data were analysed and graphed using commercially available software (SPSS 29, IBM Corp., Armonk, NY, USA and Prism 10, GraphPad Software, Boston, MA, USA). The effect of TENS or control on 6MWD and distance to the onset of claudication were assessed by a one‐way repeated‐measures analysis of variance. The effect of TENS or control on PABF, PVC, heart rate, blood pressure (systolic blood pressure (SBP), diastolic blood pressure (DBP) and mean arterial pressure (MAP)) and ABI were assessed by a two‐way repeated‐measures analysis of variance with factors of time point (baseline or post‐TENS/control) and treatment (control/no TENS, TENS15, and TENS45). Tukey's *post hoc* test was used for all pairwise comparisons. Significance was set to α ≤ 0.05. All data are reported as means ± SD and 95% confidence intervals (lower, upper). In addition to *P*‐values, effect sizes (*d*) are reported.

## 3 RESULTS

**FIGURE 1:**
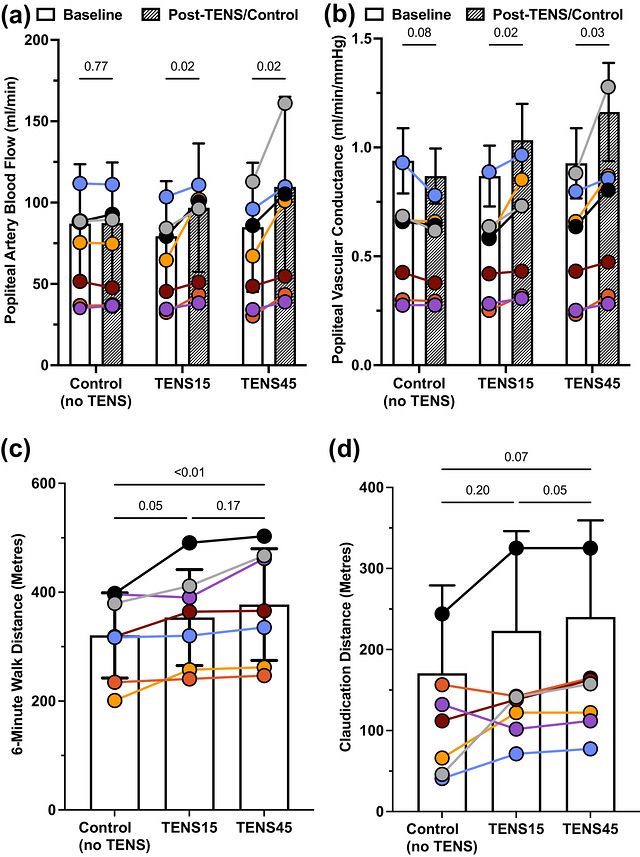
Effect of acute lower leg TENS trials on resting popliteal artery blood flow (PABF), popliteal vascular conductance (PVC), 6MWD and onset of claudication (*n* = 7). (a) PABF increased in response to both TENS15 and TENS45. (b) PVC increased in response to both TENS15 and TENS45. (c) 6MWD increased in response to both TENS15 and TENS45. (d) Distance to the onset of claudication was not statistically different between control and either TENS condition but there were medium effect sizes (see Results section for effect sizes). Data for each panel are shown as means ± SD with individual data points.

Data for PABF, PVC, 6MWD and claudication distance are shown in Figure 1. For PABF and PVC, there was a significant time point × treatment interaction effect (*P* < 0.01). Under control (no TENS) conditions, there was no difference between baseline PABF (87 ± 37 (53, 121) mL min^−1^) and post‐control PABF (87 ± 37 (53, 122) mL min^−1^) (*P *= 0.77). There was also no difference between baseline PVC (0.94 ± 0.40 (0.57, 1.31) mL min^−1^ mmHg^−1^) and post‐control PVC (0.87 ± 0.34 (0.56, 1.30) mL min^−1^ mmHg^−1^) (*P* = 0.08; *d* = 0.19). In response to TENS15, PABF increased from 79 ± 34 (48, 111) to 97 ± 40 (60, 133) mL min^−1^ (22.1%; *P* = 0.02; *d *= 0.47) and PVC increased from 0.87 ± 0.37 (0.53, 1.21) to 1.03 ± 0.44 (0.62, 1.44) mL min^−1^ mmHg^−1^ (18.9%; *P* = 0.02; *d *= 0.40). The TENS45 trial increased PABF from 85 ± 50 (48, 122) to 110 ± 55 (58, 161) mL min^−1^ (29.3%; *P* = 0.02; *d *= 0.50) and PVC from 0.93 ± 0.43 (0.53, 1.32) to 1.16 ± 0.60 (0.61, 1.72) mL min^−1^ mmHg^−1^ (25.4%; *P =* 0.03; *d *= 0.44).

Following TENS, 6MWD increased from 321 ± 78 during control to 354 ± 88 metres after TENS15 (10.3%; *P =* 0.05; *d *= 0.39) and 377 ± 103 metres after TENS45 (17.7%; *P *< 0.01; *d *= 0.61). There was no difference in 6MWD between TENS15 and TENS45 (*P *= 0.17; *d *= 0.25). Participants reported onset of claudication at 171 ± 108 metres during control compared to 223 ± 123 metres after TENS15 (*P* = 0.20; *d *= 0.45), and 240 ± 119 metres after TENS45 (*P *= 0.07; *d *= 0.61). The onset of claudication was significantly different between TENS15 and TENS45 (*P *= 0.05; *d *= 0.14).

There were no significant main or interaction effects for ABI, systolic blood pressure, or heart rate (all *P* > 0.16; Table 1). There was a main effect of time for both DBP (*P* = 0.01) and MAP (*P* = 0.04; Table 1). Although there were no main or interaction effects for ABI, HR and SBP and only a main effect of time point for DBP and MAP, haemodynamic data are shown for all conditions/time points for transparency.

**TABLE 1:** Participant haemodynamic variables.

	Baseline	Post‐TENS/Control
Resting ankle–brachial index		
Control TENS15 TENS45 Mean	0.75 ± 0.22 0.77 ± 0.20 0.75 ± 0.24 0.76 ± 0.21	0.74 ± 0.21 0.79 ± 0.20 0.77 ± 0.23 0.77 ± 0.20
Heart Rate (beats min^−1^)		
Control TENS15 TENS45 Mean	62 ± 9 61 ± 12 62 ± 8 62 ± 9	61 ± 9 61 ± 12 61 ± 8 61 ± 9
Systolic blood pressure (mmHg)		
Control TENS15 TENS45 Mean	131 ± 6 128 ± 10 130 ± 12 129 ± 10	133 ± 7 131 ± 7 131 ± 9 132 ± 7
Diastolic blood pressure (mmHg)		
Control TENS15 TENS45 Mean	74 ± 7 73 ± 9 74 ± 9 74 ± 8	75 ± 6 76 ± 8 77 ± 7 77 ± 7^*^
Mean arterial pressure (mmHg)		
Control TENS15 TENS45 Mean	93 ± 5 91 ± 8 92 ± 9 92 ± 8	95 ± 5 95 ± 7 96 ± 7 95 ± 6^*^

Values are means ± SD. Ankle–brachial index values are from most affected leg. ^*^Significant main effect of time point (*P* ≤ 0.05).

## 4 DISCUSSION

The goal of this pilot study was to determine the effect of acute lower leg TENS (for 15 or 45 min) on resting PABF, PVC and 6MWD in patients with PAD. In support of our hypothesis, both 15 and 45 min of TENS induced increases in PABF, PVC and 6MWD in participants with IC. These findings suggest as little as 15 min of lower leg TENS evokes increases in leg blood flow that may help support improved exercise tolerance in patients with PAD.

Our preliminary findings are consistent with recent studies that have shown that acute TENS can improve walking distance and onset of claudication for individuals with PAD (Labrunée et al., 2015; Seenan et al., 2016). Following TENS15 and TENS45, participants increased their 6MWD by 32.9 m (108 feet) and 56.7 m (186 feet), respectively. These increases in 6MWD compare favourably to improvement evoked by supervised exercise training, with both durations of TENS inducing moderate (≥25 m) (Gardner et al., 2018) to large (≥20/38 m) minimal clinically important differences (Gardner et al., 2018; McDermott et al., 2021). It has been suggested that TENS induces these therapeutic effects via both analgesic and vascular mechanisms.

Previous investigations suggest that TENS may promote increased perfusion via inhibition of sympathetic outflow and/or release of vasodilator substances (Debreceni et al., 1995; Jéhannin et al., 2020; Kaada, 1982). One study found that acute, low frequency TENS induced vasodilation in patients with Raynaud's syndrome and diabetic neuropathy, suggesting a direct effect of TENS on the vasculature (Kaada, 1982). Another study found that acute TENS administration reduced muscle sympathetic nerve activity in patients with heart failure, suggesting an indirect vascular effect as a decrease in sympathetic nerve activity would be expected to decrease vascular resistance and increase blood flow (Labrunée et al., 2013). In patients with PAD, chronic TENS was shown to improve walking distance and increase toe oxygen saturation, providing indirect evidence that TENS can improve vascular function and exercise capacity in this population (Debreceni et al., 1995). Moreover, chronic TENS administration may enhance production of endogenous vascular growth factors, thereby increasing skeletal muscle perfusion via augmented angiogenesis (Ellul & Gatt, 2016). Our findings extend these observations by elucidating the relationship between the duration of TENS administration and its effects on lower extremity blood flow and exercise capacity in patients with PAD. These results may inform the optimization of TENS prescription to maximize its potential therapeutic benefits in this population.

Increased blood flow to the lower extremities of individuals with PAD may subserve increased perfusion to muscles, potentially better supplying myocytes with oxygen and nutrients to improve functional mobility. However, it is important to note that recent findings indicate that skeletal muscle microvascular reactivity, rather than conduit artery blood flow or resting ABI, is the strongest predictor of 6MWD in patients with PAD (Sullivan et al., 2025). Those findings are consistent with the results of the current investigation that ABI did not significantly change in response to TENS. TENS may be implemented in a clinical and/or home setting prior to therapeutic modalities or physical activity, to potentially enhance the efficacy of these interventions. Once individuals with PAD can begin exercising for longer periods of time, they may develop further peripheral microvascular and metabolic adaptations to compensate for impaired lower extremity perfusion. Along these lines, the administration of TENS appears to preferentially recruit fatigue resistant type I muscle fibres and stimulate angiogenesis (Ellul & Gatt, 2016). Taken together, these adaptations have the potential to increase independence and improve quality of life in patients with PAD.

In addition to these vascular effects, there is substantial evidence that TENS administration can induce analgesic effects. In accordance with the gate control theory of pain (Melzack & Wall, 1965; Mendell, 2014), TENS activates afferent Aβ fibres, which stimulate inhibitory interneurons, thereby blunting transmission of nociceptive signals and altering perception of pain. Furthermore, TENS has been found to activate inhibitory pathways descending from the brainstem, which alters pain perception at the spinal level and evokes the release of endogenous opioids to further attenuate pain signals (Patel et al., 2025). Along those lines, a recent systematic review provided moderate‐certainty evidence that strong but non‐painful TENS provides a variety of participants local reductions in pain during and immediately after treatment (Johnson et al., 2022). This is particularly pertinent to the current study, as measurements of PABF, PVC and 6MWD were collected following administration of TENS. Furthermore, this may help explain the absence of a clear dose–response relationship between TENS15 and TENS45, which may implicate an analgesic versus a time‐dependent vascular effect.

There are some limitations to our preliminary investigation. First, this study was performed on a relatively small sample size, so a risk of both Type I and Type II errors exists (Johnson et al., 2022), although all participants demonstrated improvements in PABF, PVC and 6MWD following both durations of TENS. Second, although these findings demonstrate that both PABF and 6MWD increased following TENS, it is unclear the degree to which these improvements in walking capacity can be attributed to increases in leg blood flow per se. Along those lines, as noted earlier, post‐TENS PABF is an incomplete surrogate for microvascular function, which correlates with walking distance in individuals with PAD (Sullivan et al., 2025). Moreover, as discussed previously, TENS may blunt claudication symptoms via multiple analgesic mechanisms, including the gate control theory, activation of descending inhibitory pathways, and by promoting the release of endogenous opioids (Johnson et al., 2022; Patel et al., 2025). Thus, it is possible that the improvements in 6MWD in the current investigation may be largely attributed to delayed claudication resulting from a higher pain threshold, as opposed to improved muscle oxygenation via enhanced leg blood flow. It is worth noting that, in addition to the aforementioned potential mechanisms by which TENS may increase blood flow, the utilization of burst mode at an intensity to evoke skeletal muscle contraction in the current investigation likely contributed to enhanced skeletal muscle blood flow in the absence of increased metabolic demand. Additional research is needed to determine how much TENS‐induced increases in leg blood flow contribute to improved walking capacity in patients with PAD, as we cannot differentiate between the analgesic and vascular mechanisms at play in the current investigation. Finally, although we did not observe a statistically significant delay in onset of claudication following TENS, we did find moderate effect sizes (0.45 and 0.61) in this subjective measure compared to the control trial, suggesting a clinically significant improvement that may also be statistically significant with a larger sample size. Furthermore, the onset of claudication was significantly different between TENS15 and TENS45. However, given the small sample size, the current study was underpowered for secondary clinical outcomes. Therefore, further investigation is needed to determine if TENS administration has the potential to delay claudication onset and improve exercise tolerance, which in turn could promote greater engagement in physical activity in this population and disrupt the cycle of pain and deconditioning that is characteristic of PAD.

### 4.1 Perspectives and clinical significance

The current pilot study is the first to investigate the effect of different durations of TENS on both leg blood flow and walking performance in patients with PAD, finding that as little as 15 min of lower leg TENS improves post‐TENS PABF, PVC and 6MWD in these individuals. TENS is a safe and affordable intervention that can be employed in both clinic and home settings to increase exercise tolerance and, if undertaken routinely, has the potential to improve patient adherence to and efficacy of exercise prescription in these patients. The heterogeneity of PAD warrants larger clinical trials but the current data provide important proof of concept upon which to build future investigations exploring the role of TENS in treatment of this population.

## AUTHOR CONTRIBUTIONS

Conception and design of the work: Thomas K. Pellinger and Catherine B. Neighbors. Acquisition or analysis and interpretation of data for the work: Thomas K. Pellinger, Catherine B. Neighbors, Grant H. Simmons, Brett J. Wong, Joshua C. Hefta, Patrick H. Luo, Erotokritos T. Varlas, and Carl B. Suarez. Drafting the work or revising it critically for important intellectual content: Thomas K. Pellinger, Catherine B. Neighbors, Grant H. Simmons, Brett J. Wong, Joshua C. Hefta, Patrick H. Luo, Erotokritos T. Varlas, and Carl B. Suarez. All authors have read and approved the final version of this manuscript and agree to be accountable for all aspects of the work in ensuring that questions related to the accuracy or integrity of any part of the work are appropriately investigated and resolved. All persons designated as authors qualify for authorship, and all those who qualify for authorship are listed.

## CONFLICT OF INTEREST

None declared.

## GENERATIVE AI STATEMENT

No generative AI tools were used in the preparation of this manuscript.

## Data Availability

The data that support the findings of this study are available from the corresponding author upon reasonable request.
